# Cross-sectional study on the relationship between sarcopenia indicators and lung function in a community-dwelling population

**DOI:** 10.3389/fnut.2025.1721199

**Published:** 2026-01-29

**Authors:** Xiao Feng, Liuting Zheng, Yue Niu, Keyun Wang, Junqian Wang, Luying Qiao, Sifan Yang, Huanrong Li, Wang Lu, Shuang Li, Huidi Xie, Ying Zheng, Weiguang Zhang, Zhe Feng, Xiangmei Chen

**Affiliations:** 1 School of Clinical Medicine, Guangdong Pharmaceutical University, Guangzhou, China; 2 Senior Department of Nephrology, First Medical Center of Chinese PLA General Hospital, State Key Laboratory of Kidney Diseases, National Clinical Research Center for Kidney Diseases, Beijing Key Laboratory of Medical Devices and Integrated Traditional Chinese and Western Drug Development for Severe Kidney Diseases, Beijing Key Laboratory of Digital Intelligent TCM for the Prevention and Treatment of Pan-vascular Diseases, Key Disciplines of National Administration of Traditional Chinese Medicine, Innovation Team and Talents Cultivation Program of National Administration of Traditional Chinese Medicine, Beijing, China; 3 School of Basic Medical Sciences, Chengdu University of Traditional Chinese Medicine, Chengdu, China; 4 School of Clinical Medicine, Chengdu University of Traditional Chinese Medicine, Chengdu, China; 5 Department of postgraduate, Hebei North University, Zhangjiakou, China

**Keywords:** lung function, muscle strength, physical performance, preserved ratio impaired spirometry, sarcopenia, skeletal muscle mass index

## Abstract

**Background:**

With advancing age, multiple systems, including the muscular and respiratory systems, undergo degenerative changes. While the relationship between sarcopenia and lung function is well-established in patients with respiratory diseases, evidence remains limited in the general population. Therefore, this study aimed to investigate the relationship between sarcopenia indicators and lung function in a community-dwelling population.

**Methods:**

Basic information, laboratory biochemical parameters, handgrip strength (HGS), gait speed (GS), five times sit-to-stand test (5STS) time, skeletal muscle mass index (SMI) and lung function parameters, were collected from a community-dwelling population in Beijing, China. Linear regression analysis was employed to investigate the relationship between sarcopenia indicators and lung function parameters, and logistic regression analysis was utilized to examine their association with lung function statuses.

**Results:**

A total of 2,526 community volunteers were enrolled and divided into three groups based on lung function: normal spirometry (*n* = 2,032), preserved ratio impaired spirometry (PRISm) (*n* = 231), and obstructive spirometry (*n* = 273). After adjusting for covariates, linear regression analysis revealed that HGS, GS, and SMI were positively correlated with forced expiratory volume in one second (FEV1) and forced vital capacity (FVC), while 5STS was negatively correlated with FEV1 and FEV1/FVC ratio. Logistic regression analysis revealed that lower HGS and longer 5STS time were associated with a higher risk of PRISm, while slower GS, longer 5STS time were associated with an increased risk of obstructive spirometry. Sarcopenia was associated with both PRISm risk and obstructive spirometry risk.

**Conclusion:**

This study demonstrates that sarcopenia indicators are closely associated with lung function in community-dwelling individuals. As a condition that significantly impacts healthy aging and is linked to both quality of life and longevity, enhancing the early identification and management of sarcopenia within community settings may yield multi-organ benefits.

## 1 Introduction

Sarcopenia is an age-related syndrome characterized by a decline in skeletal muscle mass, muscle strength, and physical function. Its risk increases with age (1, 2) and is closely linked to falls, disability, reduced quality of life, and increased mortality (3, 4). Underlying mechanisms involve multiple factors, including low physical activity, malnutrition, and chronic inflammation.

Lungs reach maturity between ages 20–25 (5). Subsequently, with advancing age, respiratory muscle strength and lung tissue elasticity decline, impairing gas exchange and leading to reduced lung function parameters such as forced vital capacity (FVC) and forced expiratory volume in one second (FEV1) (6). This decline in lung function represents not only a common physiological change during aging but also the pathological foundation for numerous respiratory diseases. Chronic obstructive pulmonary disease (COPD), a prevalent respiratory condition, has a remarkably high prevalence of 13.7% among individuals aged 40 and older (7). With the accelerating pace of population aging, a growing number of individuals are likely to be in the preclinical stage of COPD known as preserved ratio impaired spirometry (PRISm). Approximately 32.6% of individuals with PRISm progress to COPD within 5 years, accompanied by heightened risks of cardiovascular disease and mortality (8, 9).

In patients with respiratory diseases, including COPD, asthma, and interstitial pneumonia, sarcopenia is closely associated with lung function. Compared to those without sarcopenia, these patients face a higher risk of adverse events and poorer prognosis (10–12). One study demonstrated that during follow-up, sarcopenia-related declines in physical function correlated with worsening lung function (13). These findings suggest that early identification and management of sarcopenia play a critical role in preserving lung function and promoting respiratory health.

The assessment of sarcopenia typically involves three domains: muscle strength, muscle mass, and physical function. Handgrip strength (HGS) is a commonly used indicator of muscle strength. Studies have shown that in patients with respiratory diseases, such as COPD, HGS and muscle mass are positively correlated with FVC and FEV1 (14, 15). Meanwhile, physical function indicators like slower gait speed (GS) are associated with increased risks of hospitalization rates and mortality (16). However, the relevant evidence remains limited in the general population or among individuals in the preclinical stage. Most existing studies have either treated sarcopenia as an overall diagnosis or focused on single indicators, overlooking its multidimensional characteristics as a clinical syndrome. In fact, different assessment indicators for sarcopenia may exhibit varying sensitivity and specificity in reflecting changes in lung function (17), leaving a research gap in systematically evaluating the relationship between these indicators and different lung function statuses.

Against this backdrop, this study comprehensively evaluates the relationship between individual components of sarcopenia and lung function in a community-dwelling population in Beijing, China. The aim is to provide evidence for understanding their relationship and for early intervention strategies.

## 2 Methods

### 2.1 Study design and participants

This study was conducted from June 2024 to December 2024, initially recruiting volunteers from communities in Beijing, China, based on the following exclusion criteria: (1) Age < 18 years; (2) Diagnosed with respiratory diseases such as chronic obstructive pulmonary disease, asthma, or bronchiectasis; (3) Diagnosed with rheumatic diseases such as fractures or rheumatoid arthritis; (4) Diagnosed with any of the following conditions: diabetes, hypertension, chronic kidney disease, liver cirrhosis, stroke, myocardial infarction, malignant hematologic disorders, or malignant tumors; (5) Presence of medical implants such as cardiac stents, pacemakers, metal plates, or pins, rendering them unsuitable for BIA measurement; (6) Conditions potentially affecting body composition test accuracy, such as edema; (7) Individuals unwilling to cooperate with indicator testing or with missing sample data. Participants newly identified with hypertension, abnormal blood glucose, or abnormal blood lipids during the study period but not previously diagnosed will still be retained for inclusion in this study. Ultimately, 2,526 volunteers were enrolled in the study. This research was conducted in accordance with the Declaration of Helsinki, and the study protocol was approved by the Ethics Committee. All volunteers signed informed consent forms prior to participation.

### 2.2 Data collection

Demographic information, including sex, age, and smoking status, was collected from the participants. Height and weight were measured using an anthropoid measuring device (Seca 213, Hamburg, Germany) and a digital scale (Omron HN-289-BK, Kyoto, Japan), respectively. Body mass index (BMI) was calculated as weight (kg) divided by height squared (m^2^). With the participant seated at rest in a calm state, blood pressure was measured three times on the dominant arm using an automated electronic device (Omron HEM-757, Kyoto, Japan), with a 1-min rest between measurements. The mean systolic and diastolic blood pressures were recorded. Hypertension was defined as systolic blood pressure ≥140 mmHg and/or diastolic blood pressure ≥90 mmHg.

Fasting venous blood samples were collected from participants for biochemical analysis. Blood biochemistry was performed using the Roche Cobas 8000 automated biochemical analyzer with the C701 module (Roche Diagnostics, Mannheim, Germany), employing Roche original reagents and spectrophotometry. Complete blood counts and hemoglobin analysis were performed using the Sysmex XN-9000 automated hematology analyzer (Kobe, Japan) for complete blood cell counts and hemoglobin analysis. Parameters recorded included hemoglobin (Hb), albumin (Alb), fasting plasma glucose (FPG), triglycerides (TG), total cholesterol (TC) and serum creatinine (SCr). Estimated glomerular filtration rate (eGFR) was calculated using the Chronic Kidney Disease Epidemiology Collaboration (CKD-EPI) equation based on serum creatinine. Diabetes was defined as glycated hemoglobin >6.0% and/or fasting plasma glucose ≥7.0 mmol/L.

Pre-bronchodilator spirometry was performed using the Jäger MasterScreen Pneumo device (CareFusion, Hoechberg, Germany). The operational procedures and quality control standards adhered to the American Thoracic Society and European Respiratory Society (ATS-ERS) guidelines (18, 19), with reference equations for predicted lung function values based on the ECSC 1993 equation (20). All pulmonary function tests were conducted between 8:00 a.m. and 12:00 p.m. Analysis utilized collected lung function parameters, including FVC, FEV1, the FEV1/FVC ratio, and the percentage of FEV1 to predicted FEV1 (FEV1%). PRISm was defined as a FEV1% < 80% and a FEV1/FVC ratio ≥0.70 (21). Obstructive spirometry was defined as a FEV1/FVC ratio < 0.70 according to the Global Initiative for Chronic Obstructive Lung Disease (GOLD) (22). Normal spirometry was defined as a FEV1% >80% and a FEV1/FVC ratio ≥0.70.

Handgrip strength (HGS) was measured three times using a Jamar dynamometer (Sammons Preston Rolyan, Bolingbrook, IL) on the participant's dominant hand. The mean of the three HGS measurements was used for analysis. All participants performed two sets of the five-times sit-to-stand test (5STS), separated by a 5-min rest period, with the best score from each set used for analysis. Participants were instructed to walk 6 meters at their usual pace. The time taken was recorded, and the gait speed (GS) was calculated for analysis. Muscle mass was measured using the InBody770 body composition analyzer (BioSpace, Seoul, Korea). Skeletal muscle mass index (SMI) was calculated as muscle mass (kg) divided by height squared (m^2^). The diagnosis of sarcopenia is based on the definition established by the Asian Working Group for Sarcopenia (AWGS) in 2019 (23).

### 2.3 Statistical methods

Participants were divided into three groups based on lung function: normal, PRISm, and obstructive. Specific grouping criteria were described in the previous section. Continuous variables are presented as mean ± standard deviation (SD). Categorical variables are expressed as frequency (*n*) and percentage (%). Differences between two distinct groups were compared using *t*-tests for continuous data and chi-square tests for categorical data. Linear regression was employed to analyze the relationship between sarcopenia indicators and lung function parameters (FEV1, FVC, FEV1/FVC). Multivariate logistic regression was used to analyze risk factors for PRISm and obstructive spirometry. To facilitate comparisons between different indicators, continuous variables were standardized (*z*-scores) in the logistic model. Additionally, sarcopenia indicators were grouped by quartiles (Q1–Q4), and their trend relationships with lung function status were assessed in logistic regression. Covariates included age, sex, BMI, hypertension, diabetes, smoking, TC, TG, eGFR and other potential confounding factors. Collinearity was assessed using variance inflation factors (VIF), while residual plots evaluated the normality and homogeneity of variance of the linear model. Results are presented as regression coefficients (β), odds ratios (OR), and their 95% confidence intervals (CI). A two-sided *p*-value < 0.05 was considered statistically significant. Data were analyzed using SPSS 26.0 (IBM, Armonk, NY, USA).

## 3 Results

### 3.1 Participant characteristics

A total of 2,526 participants were included in this study. The mean age was 53.33 years, and 58.91% were female. Participants were divided into three groups based on lung function results: 231 in the PRISm group, 273 in the obstructive spirometry group, and 2032 in the normal spirometry group. Compared with participants with normal spirometry, participants in the PRISm group had a higher proportion of females, hypertension, and diabetes, along with lower HGS, slower GS, lower SMI, and longer 5STS time. The obstructive spirometry group was older, and had a higher proportion of males, smoking, hypertension, and diabetes, as well as lower BMI, lower eGFR, longer 5STS time and slower GS. Both groups demonstrated higher rates of sarcopenia compared to the normal spirometry group. Specific participant characteristics are shown in Table 1.

**Table 1:** Characteristics of participants.

**Characteristics**	**Total**	**Normal spirometry**	**PRISm**	**Obstructive spirometry**	***P*-value (PRISm vs. Normal)**	***P*-value (obstructive vs. normal)**
*n*	2,536	2,032	231	273		
Age, years	53.33 ± 10.68	52.74 ± 10.32	53.01 ± 11.45	58.03 ± 11.51	0.707	< 0.001
Height, cm	165.25 ± 7.67	165.13 ± 7.64	163.97 ± 7.06	167.23 ± 8.07	0.020	< 0.001
Weight, kg	67.03 ± 11.91	67.07 ± 11.93	66.40 ± 11.93	67.63 ± 11.78	0.417	0.802
BMI, kg/m^2^	24.45 ± 3.37	24.50 ± 3.37	24.61 ± 3.54	23.95 ± 3.15	0.644	0.011
Sex *n* (%)					0.130	< 0.001
Male	1,042 (41.09%)	817 (40.21%)	81 (35.06%)	144 (52.75%)		
Female	1,494 (58.91%)	1,215 (59.79%)	150 (64.94%)	129 (47.25%)		
Smoker *n* (%)	595 (23.46%)	458 (22.54%)	55 (23.81%)	82 (30.04%)	0.662	0.006
Hypertension *n* (%)	672 (26.50%)	508 (25.00%)	74 (32.03%)	90 (32.97%)	0.020	0.005
Diabetes *n* (%)	476 (18.77%)	355 (17.47%)	55 (23.81%)	66 (24.18%)	0.018	0.007
Hb, g/L	141.78 ± 15.62	141.55 ± 15.67	140.93 ± 16.61	144.18 ± 14.09	0.569	0.008
Alb, g/L	47.27 ± 2.62	47.34 ± 2.53	47.40 ± 2.25	46.68 ± 3.36	0.739	< 0.001
eGFR, mL/min/1.73 m^2^	92.02 ± 13.70	92.42 ± 13.22	93.72 ± 15.18	87.63 ± 15.11	0.213	< 0.001
TC, mmol/L	5.02 ± 1.00	5.03 ± 1.01	4.99 ± 0.96	4.95 ± 1.00	0.509	0.177
TG, mmol/L	1.51 ± 1.29	1.52 ± 1.36	1.46 ± 1.04	1.41 ± 0.87	0.497	0.180
FPG, mmol/L	5.51 ± 1.21	5.47 ± 1.15	5.72 ± 1.65	5.57 ± 1.22	0.027	0.224
HbA1C, %	5.79 ± 0.72	5.77 ± 0.68	5.93 ± 0.89	5.85 ± 0.79	0.009	0.133
FEV1, L	2.62 ± 0.68	2.76 ± 0.63	2.01 ± 0.48	2.12 ± 0.65	< 0.001	< 0.001
FEV1 predicted, L	2.80 ± 0.61	2.81 ± 0.61	2.75 ± 0.57	2.80 ± 0.62	0.131	0.912
FEV1, %predicted	93.93 ± 15.61	98.73 ± 11.69	72.76 ± 6.43	76.11 ± 18.54	< 0.001	< 0.001
FVC, L	3.35 ± 0.83	3.43 ± 0.79	2.56 ± 0.66	3.41 ± 0.89	< 0.001	0.685
FEV1/FVC, %	78.59 ± 8.29	80.74 ± 5.24	79.18 ± 6.28	62.11 ± 9.70	< 0.001	< 0.001
HGS, kg	32.56 ± 10.96	32.90 ± 11.09	29.66 ± 9.50	32.51 ± 10.83	< 0.001	0.587
5STS, s	6.74 ± 1.88	6.60 ± 1.80	7.09 ± 1.80	7.56 ± 2.26	< 0.001	< 0.001
GS, m/s	1.16 ± 0.17	1.17 ± 0.17	1.13 ± 0.17	1.11 ± 0.18	< 0.001	< 0.001
SMI, kg/m^2^	7.02 ± 1.02	7.03 ± 1.02	6.89 ± 1.06	7.09 ± 1.00	0.049	0.327
Sarcopenia, *n* (%)	69 (2.72%)	42 (2.07%)	11 (4.76%)	16 (5.86%)	0.010	< 0.001

All values are presented as mean ± SD or *n* (%). All *P*-values were calculated with a two-sided significance level of 0.05. SD, standard deviation; PRISm, preserved ratio impaired spirometry; BMI, body mass index; Hb, hemoglobin; Alb, albumin; eGFR, estimated glomerular filtration rate; TC, total cholesterol; TG, triglycerides; FPG, fasting plasma glucose; HbA1C, hemoglobin A1C; FEV1, forced expiratory volume in 1 s; FVC, forced vital capacity; HGS, handgrip strength; 5STS, five times sit-to-stand test; GS, gait speed; SMI, skeletal muscle mass index.

### 3.2 Relationship between sarcopenia indicators and lung function parameters

Linear regression analysis was used to evaluate the relationship between sarcopenia indicators (HGS, GS, 5STS time, and SMI) and lung function parameters (FEV1, FVC, FEV1/FVC), with results shown in Table 2. HGS showed significant positive correlations with FEV1 and FVC, which remained consistent after adjusting for all covariates: FEV1 (β = 0.014, 95% CI: 0.012, 0.017) and FVC (β = 0.019, 95% CI: 0.016, 0.022). A significant negative correlation was observed between 5STS time and FEV1, FVC, and FEV1/FVC. After adjusting for covariates, significant negative correlations remained with FEV1 and FEV1/FVC: FEV1 (β = −0.018, 95% CI: −0.028, −0.008), FEV1/FVC (β = −0.486, 95% CI: −0.668, −0.304). GS was significantly positively correlated with FEV1, FVC, and FEV1/FVC. These correlations persisted after covariate adjustment: FEV1 (β = 0.344, 95% CI: 0.238, 0.450), FVC (β = 0.344, 95% CI: 0.210, 0.459), FEV1/FVC (β = 2.493, 95% CI: 0.547, 4.411). SMI was significantly positively correlated with FEV1 and FVC, and significantly negatively correlated with FEV1/FVC. These associations remained consistent after adjustment for covariates: FEV1 (β = 0.265, 95% CI: 0.223, 0.307), FVC (β = 0.390, 95% CI: 0.342, 0.439), FEV1/FVC (β = −1.265, 95% CI: −2.035, −0.470).

**Table 2:** Associations of sarcopenia-related indicators with lung function parameters in multivariable linear regression models.

**Sarcopenia indicators**	**FEV1**				**FVC**				**FEV1/FVC**			
	**Unadjusted**		**Adjusted**		**Unadjusted**		**Adjusted**		**Unadjusted**		**Adjusted**	
	β **(95%CI)**	***P*** **-value**	β **(95%CI)**	***P*** **-value**	β **(95%CI)**	***P*** **-value**	β **(95%CI)**	***P*** **-value**	β **(95%CI)**	***P*** **-value**	**B (95%CI)**	***P*** **-value**
HGS	0.039 (0.037, 0.041)	< 0.001	0.014 (0.012, 0.017)	< 0.001	0.051 (0.049, 0.053)	< 0.001	0.019 (0.016, 0.022)	< 0.001	−0.028 (−0.057, 0.002)	0.064	−0.005 (−0.050, 0.040)	0.835
5STS	−0.077 (−0.091, −0.064)	< 0.001	−0.018 (−0.028, −0.008)	< 0.001	−0.069 (−0.086, −0.052)	< 0.001	−0.018 (−0.014, 0.010)	0.754	−0.671 (−0.841, −0.501)	< 0.001	−0.486 (−0.668, −0.304)	< 0.001
GS	0.867 (0.713, 1.020)	< 0.001	0.344 (0.238, 0.450)	< 0.001	0.950 (0.761, 1.138)	< 0.001	0.334 (0.210, 0.459)	< 0.001	3.572 (1.659, 5.485)	< 0.001	2.493 (0.547, 4.411)	0.011
SMI	0.373 (0.352, 0.395)	< 0.001	0.265 (0.223, 0.307)	< 0.001	0.495 (0.470, 0.520)	< 0.001	0.390 (0.342, 0.439)	< 0.001	−0.485 (−0.800, −0.169)	0.003	−1.265 (−2.035, −0.470)	0.002

Unadjusted model was adjusted for no covariates; Adjusted model was adjusted for age, sex, BMI, smoker, hypertension, diabetes, Hb, Alb, eGFR, TC, TG. CI, confidence interval; BMI, body mass index; Hb, hemoglobin; Alb, albumin; eGFR, estimated glomerular filtration rate; TC, total cholesterol; TG, triglycerides; FEV1, forced expiratory volume in 1 s; FVC, forced vital capacity; HGS, handgrip strength; 5STS, five times sit-to-stand test; GS, gait speed; SMI, skeletal muscle mass index.

### 3.3 Relationship between sarcopenia indicators and lung function statuses

Lung function status served as the dependent variable, with continuous variables standardized to ensure consistency. Using the normal spirometry group as the reference, multivariate logistic regression analysis was performed, incorporating HGS, GS, 5STS time, SMI, and covariates. The results are presented in Table 3. Compared with females, males have a higher risk of PRISm (OR: 1.942, 95% CI: 1.003, 3.757). HGS (OR per SD increase: 0.617, 95% CI: 0.482, 0.789) and 5STS time (OR per SD increase: 1.225, 95% CI: 1.057, 1.418) were associated with PRISm risk, while other measures showed no significant association. Slower GS (OR per SD increase: 0.792, 95% CI: 0.689, 0.914) and longer 5STS time (OR per SD increase: 1.319, 95% CI: 1.156, 1.505) were both associated with increased risk of obstructive spirometry. Additionally, older age (OR per SD increase: 1.215, 95% CI: 1.011, 1.462), lower BMI (OR per SD increase: 0.641, 95% CI: 0.510, 0.806), higher Hb (OR per SD increase: 1.270, 95% CI: 1.043, 1.546), and lower Alb (OR per SD increase: 0.836, 95% CI: 0.745, 0.939) were associated with the risk of obstructive spirometry.

**Table 3:** Odds ratios of sarcopenia-related indicators for lung function statuses in multivariable standardized logistic regression analysis.

**Variables**	**Normal (*n* = 2,032)**	**PRISm (*****n*** = **231)**		**Obstructive (*****n*** = **273)**	
		**OR (95%CI)**	***P*** **-value**	**OR (95%CI)**	***P*** **-value**
Age	Reference	0.822 (0.676–1.000)	0.050	1.215 (1.011–1.462)	0.038
Sex					
Male		1.942 (1.003–3.757)	0.049	1.429 (0.781–2.616)	0.247
Female		Reference		Reference	
BMI	Reference	1.182 (0.934–1.494)	0.164	0.641 (0.510–0.806)	< 0.001
Smoker	Reference	1.355 (0.917–2.002)	0.127	1.091 (0.774–1.538)	0.618
Hypertension	Reference	1.389 (1.000–1.930)	0.050	1.219 (0.900–1.652)	0.200
Diabetes	Reference	1.331 (0.935–1.896)	0.112	1.253 (0.900–1.745)	0.181
Hb	Reference	1.055 (0.867–1.283)	0.595	1.270 (1.043–1.546)	0.017
Alb	Reference	1.074 (0.904–1.276)	0.419	0.836 (0.745–0.939)	0.002
eGFR	Reference	1.065 (0.890–1.275)	0.493	0.900 (0.767–1.056)	0.196
TC	Reference	0.967 (0.831–1.125)	0.660	0.956 (0.829–1.103)	0.541
TG	Reference	0.924 (0.770–1.108)	0.392	0.872 (0.720–1.057)	0.162
HGS	Reference	0.617 (0.482–0.789)	< 0.001	0.841 (0.680–1.039)	0.108
5STS	Reference	1.225 (1.057–1.418)	0.007	1.319 (1.156–1.505)	< 0.001
GS	Reference	0.880 (0.756–1.025)	0.100	0.792 (0.686–0.914)	0.001
SMI	Reference	0.715 (0.496–1.030)	0.071	1.244 (0.897–1.724)	0.191

All continuous predictors were standardized; ORs represent the risk change per 1-SD increase. OR, odds ratios; CI, confidence interval; SD, standard deviation; PRISm, preserved ratio impaired spirometry; BMI, body mass index; Hb, hemoglobin; Alb, albumin; eGFR, estimated glomerular filtration rate; TC, total cholesterol; TG, triglycerides; HGS, handgrip strength; 5STS, five times sit-to-stand test; GS, gait speed; SMI, skeletal muscle mass index.

To further validate the relationship between sarcopenia indicators and lung function statuses, we analyzed HGS, 5STS time, GS, and SMI by quartiles (Supplementary Table 1). The results were generally consistent with those from the analysis of standardized continuous variables. Additionally, inclusion of sarcopenia in the logistic regression analysis (Supplementary Table 2) revealed that sarcopenia was significantly associated with increased risk of PRISm (OR: 2.448, 95% CI: 1.208, 4.960) and obstructive spirometry (OR: 1.945, 95% CI: 1.030, 3.672).

## 4 Discussion

This study, based on a community-dwelling population in Beijing, China, examined the relationship between multiple sarcopenia indicators and lung function parameters, as well as different lung function statuses. We found that sarcopenia indicators, including HGS, GS, 5STS time, and SMI, were significantly correlated with lung function parameters such as FEV1 and FVC. Further analysis indicated that lower HGS and longer 5STS time were associated with higher PRISm risk. Slower GS, longer 5STS time, lower BMI and Alb levels were associated with increased risk of obstructive spirometry. Collectively, these findings suggest that sarcopenia indicators may hold significant value in the early identification of declining and abnormal lung function statuses.

Handgrip strength serves as an indicator of overall muscle strength, and its decline may reflect weakened respiratory muscle strength, leading to reduced ventilatory reserve and increased risk of respiratory mortality (24). In linear analyses, we found that grip strength was positively correlated with both FEV1 and FVC, both before and after adjusting for covariates. This finding is consistent with the results observed by Son et al. (25) and Martinez et al. (14) in elderly Korean women and COPD patients. Our study further revealed an independent association between lower HGS and increased PRISm risk. Given that PRISm is considered a transitional state between normal lung function and COPD, we hypothesize that the decline in whole-body muscle strength represented by HGS may serve as a sensitive indicator of early-stage lung function deterioration and that grip strength training may potentially improve lung function (26).

The five-times sit-to-stand test reflects limb muscle strength and function, but research on the relationship between 5STS and lung function remains limited and inconclusive. Choi et al. (27) reported no association between 5STS time and lung function parameters in Korean adults, whereas Landi et al. (13) observed significant correlations between 5STS time and both FEV1 and FVC in Italian adults. Our study found a significantly negative correlation between 5STS time and both FEV1 and FEV1/FVC, adding to the evidence on the relationship between 5STS and lung function. Furthermore, we observed that longer 5STS time was associated with an increased risk of both PRISm and obstructive spirometry. This suggests that declines in lower limb muscle strength and functional capacity may play a role across different stages of lung function impairment, serving not only as an indicator of early decline but also showing a strong association with more advanced states of airflow limitation.

The relationship between gait speed and lung function has been well-supported by research evidence, particularly in patients with COPD. Ilgin et al. (28) observed significant associations between GS and lung function in patients with COPD. Mendelian randomization findings by Wang et al. (16) further suggest that slower GS increases the risk of developing COPD, accelerates lung function decline, and raises hospitalization risk. Our study confirmed the association between GS and lung function parameters in a community population and found that slower GS was associated with the risk of obstructive spirometry. In COPD patients, airflow limitation causes dyspnea during physical activity, thereby restricting gait speed. We observed this association in community-dwelling individuals without diagnosed respiratory disease. Combined with previous studies, we propose that gait speed may serve as a sensitive indicator of early-stage lung function decline. The decline in gait speed may occur before clinical diagnosis of COPD and is closely associated with the onset and progression of obstructive ventilatory impairment. The specific mechanisms and temporal sequence require further investigation.

The skeletal muscle mass index is an indicator of appendicular skeletal muscle mass. Our study found that SMI is positively correlated with FEV1 and FVC. This finding is consistent with the results of studies by Jeon et al. (29) and Park et al. (30) in Korean adult populations. The results of our study showed a negative correlation between SMI and FEV1/FVC ratio. This might be attributed to the fact that individuals with a higher SMI tend to have a greater absolute FVC value, leading to a lower FEV1/FVC ratio when calculated. In logistic regression analysis, SMI showed no significant association with either PRISm or obstructive spirometry. This suggests that SMI plays a relatively limited role in identifying abnormal lung function statuses, functional indicators (such as 5STS time and GS) may offer more sensitive assessment basis for early lung function impairment.

Additionally, our study observed that lower BMI and Alb levels were associated with increased risk of obstructive spirometry. This suggests that obstructive spirometry abnormalities are not merely localized respiratory issues, but are also accompanied by changes in metabolic and nutritional status. Changes in these parameters may reflect or coincide with the onset of malnutrition, future interventions targeting declining lung function in community and clinical settings need to incorporate nutritional management. Furthermore, based on the AWGS 2019 diagnostic criteria, we found significant associations between sarcopenia and both PRISm and obstructive spirometry. This suggests that reduced overall muscle mass and functional decline may collectively reflect the presence of abnormal lung function.

This study explored the relationship between various sarcopenia indicators and lung function parameters and statuses, expanding the scope of existing research and providing more comprehensive evidence. The study subjects were a community-dwelling population covering middle-aged, young, and elderly groups. This makes the results more reflective of the preclinical-stage relationships between lung function and sarcopenia indicators, laying a foundation for future interventions.

However, this study has certain limitations. Its cross-sectional design cannot establish a temporal relationship between changes in sarcopenia indicators and respiratory impairment, which requires further clarification through longitudinal research. In terms of research methodology, although we adjusted for multiple confounding factors, unmeasured variables, such as detailed dietary patterns and types of physical activity, may still influence the results.

In summary, this study found that sarcopenia and its related indicators are closely associated with lung function and show differences across different lung function statuses. HGS and 5STS time were associated with increased PRISM risk, while GS, 5STS time, and malnutrition status were associated with an increased risk of obstructive spirometry. Sarcopenia itself is associated with both types of lung function abnormalities. These findings suggest that assessing sarcopenia in community populations and along with timely muscle strength evaluation and functional training may aid in the early identification and management of abnormal lung function, thereby reducing the future burden of respiratory diseases.

## Data Availability

The datasets presented in this article are not readily available as subsequent analyses in other aspects are ongoing. Requests to access the datasets should be directed to the corresponding authors.
