# Correction: Cross-sectional study on the relationship between sarcopenia indicators and lung function in a community-dwelling population

**DOI:** 10.3389/fnut.2026.1898988

**Published:** 2026-06-17

**Authors:** Xiao Feng, Liuting Zheng, Yue Niu, Keyun Wang, Junqian Wang, Luying Qiao, Sifan Yang, Huanrong Li, Wang Lu, Shuang Li, Huidi Xie, Ying Zheng, Weiguang Zhang, Zhe Feng, Xiangmei Chen

**Affiliations:** 1 School of Clinical Medicine, Guangdong Pharmaceutical University, Guangzhou, China; 2 Senior Department of Nephrology, First Medical Center of Chinese PLA General Hospital, State Key Laboratory of Kidney Diseases, National Clinical Research Center for Kidney Diseases, Beijing Key Laboratory of Medical Devices and Integrated Traditional Chinese and Western Drug Development for Severe Kidney Diseases, Beijing Key Laboratory of Digital Intelligent TCM for the Prevention and Treatment of Pan-vascular Diseases, Key Disciplines of National Administration of Traditional Chinese Medicine, Innovation Team and Talents Cultivation Program of National Administration of Traditional Chinese Medicine, Beijing, China; 3 School of Basic Medical Sciences, Chengdu University of Traditional Chinese Medicine, Chengdu, China; 4 School of Clinical Medicine, Chengdu University of Traditional Chinese Medicine, Chengdu, China; 5 Department of postgraduate, Hebei North University, Zhangjiakou, China

**Keywords:** lung function, muscle strength, physical performance, preserved ratio impaired spirometry, sarcopenia, skeletal muscle mass index

Affiliation [School of Clinical Medicine, Guangdong Pharmaceutical University, Guangzhou, China] was omitted from the paper. This affiliation has now been added for author(s) [Xiangmei Chen].

The original version of this article has been updated.

