# Supplementary material for: Cross-sectional study on the relationship between sarcopenia indicators and lung function in a community-dwelling population
Source: Front Nutr. 2026 Jan 29;12:1721199. doi: 10.3389/fnut.2025.1721199 (PMC12908173; doi:10.3389/fnut.2025.1721199)
Supplement: Supplementary file 1 [file Table_1.docx]

**Supplementary Table 1.** Odds ratios of sarcopenia-related indicators quartiles for lung function statuses in multivariable logistic regression analysis

| Variables | Normal  (n=2032) | PRISm  (n=231) | | Obstructive  (n=273) | |
| --- | --- | --- | --- | --- | --- |
|  |  | OR (95%CI) | P value | OR (95%CI) | P value |
| Age | Reference | 0.813 (0.671–0.986) | 0.036 | 1.235 (1.029–1.482) | 0.024 |
| Sex |  |  |  |  |  |
| Male |  | 1.935 (0.997–3.754) | 0.051 | 1.585 (0.835–3.006) | 0.159 |
| Female |  | Reference |  | Reference |  |
| BMI | Reference | 1.122 (0.923–1.363) | 0.247 | 0.655 (0.534–0.803) | <0.001 |
| Smoker | Reference | 1.313 (0.885–1.947) | 0.176 | 1.112 (0.790–1.566) | 0.542 |
| Hypertension | Reference | 1.401 (1.006–1.949) | 0.046 | 1.233 (0.911–1.668) | 0.175 |
| Diabetes | Reference | 1.319 (0.925–1.881) | 0.127 | 1.258 (0.904–1.750) | 0.173 |
| Hb | Reference | 1.070 (0.881–1.300) | 0.493 | 1.264 (1.038–1.540) | 0.020 |
| Alb | Reference | 1.068 (0.897–1.272) | 0.458 | 0.835 (0.743–0.939) | 0.003 |
| eGFR | Reference | 1.068 (0.894–1.276) | 0.470 | 0.896 (0.764–1.051) | 0.178 |
| TC | Reference | 0.973 (0.835–1.134) | 0.727 | 0.956 (0.829–1.103) | 0.538 |
| TG | Reference | 0.909 (0.756–1.094) | 0.313 | 0.868 (0.714–1.054) | 0.154 |
| HGS | Reference |  | 0.739* |  | 0.377* |
| Q1 |  | 3.491 (1.820–6.697) | <0.001 | 1.850 (1.018–3.360) | 0.044 |
| Q2 |  | 2.789 (1.521–5.115) | 0.001 | 1.383 (0.804–2.380) | 0.241 |
| Q3 |  | 1.617 (0.988–2.646) | 0.056 | 1.008 (0.678–1.497) | 0.970 |
| Q4 |  | Reference |  | Reference |  |
| 5STS | Reference |  | <0.001* |  | <0.001* |
| Q1 |  | Reference |  | Reference |  |
| Q2 |  | 1.463 (0.934–2.291) | 0.096 | 1.244 (0.806–1.921) | 0.324 |
| Q3 |  | 1.923 (1.237–2.991) | 0.004 | 1.540 (1.007–2.357) | 0.046 |
| Q4 |  | 2.304 (1.461–3.635) | <0.001 | 2.164 (1.415–3.307) | <0.001 |
| GS | Reference |  | 0.021* |  | 0.485* |
| Q1 |  | 1.354 (0.911–2.013) | 0.134 | 1.583 (1.067–2.347) | 0.022 |
| Q2 |  | 0.910 (0.603–1.374) | 0.654 | 1.351 (0.912–2.002) | 0.133 |
| Q3 |  | 0.686 (0.443–1.062) | 0.091 | 0.967 (0.634–1.474) | 0.875 |
| Q4 |  | Reference |  | Reference |  |
| SMI | Reference |  | 0.819* |  | 0.993* |
| Q1 |  | 2.274 (0.986–5.242) | 0.054 | 0.568 (0.263–1.229) | 0.151 |
| Q2 |  | 1.733 (0.847–3.547) | 0.132 | 0.691 (0.364–1.311) | 0.258 |
| Q3 |  | 1.504 (0.895–2.528) | 0.123 | 0.865 (0.569–1.315) | 0.498 |
| Q4 |  | Reference |  | Reference |  |

All continuous predictors were standardized; ORs represent the risk change per 1-SD increase; ^*^*p* for trend.

Abbreviations: OR, odds ratios; CI, confidence interval; SD, standard deviation; PRISm, preserved ratio impaired spirometry; BMI, body mass index; Hb, hemoglobin; Alb, albumin; eGFR, estimated glomerular filtration rate; TC, total cholesterol; TG, triglycerides; HGS, handgrip strength; 5STS, five times sit-to-stand test; GS, gait speed; SMI, skeletal muscle mass index.
