# Supplementary material for: Cross-sectional study on the relationship between sarcopenia indicators and lung function in a community-dwelling population
Source: Front Nutr. 2026 Jan 29;12:1721199. doi: 10.3389/fnut.2025.1721199 (PMC12908173; doi:10.3389/fnut.2025.1721199)
Supplement: Supplementary file 2 [file Table_2.docx]

**Supplementary Table 2.** Odds ratios of sarcopenia for lung function statuses in multivariable logistic regression analysis

|  | Normal  (n=2032) | PRISm  (n=231) | | Obstructive  (n=273) | |
| --- | --- | --- | --- | --- | --- |
|  |  | OR (95%CI) | P value | OR (95%CI) | P value |
| Age | Reference | 1.033 (0.861–1.238) | 0.727 | 1.446 (1.222–1.710) | <0.001 |
| Sex |  |  |  |  |  |
| Male |  | 0.650 (0.425–0.995) | 0.047 | 1.410 (0.961–2.069) | 0.079 |
| Female |  | Reference |  | Reference |  |
| BMI | Reference | 1.033 (0.888–1.202) | 0.671 | 0.763 (0.650–0.896) | 0.001 |
| Smoker | Reference | 1.308 (0.888–1.927) | 0.174 | 1.176 (0.840–1.645) | 0.345 |
| Hypertension | Reference | 1.408 (1.017–1.949) | 0.039 | 1.264 (0.937–1.705) | 0.125 |
| Diabetes | Reference | 1.400 (0.988–1.984) | 0.059 | 1.317 (0.951–1.825) | 0.097 |
| Hb | Reference | 1.037 (0.854–1.259) | 0.717 | 1.207 (0.994–1.466) | 0.057 |
| Alb | Reference | 1.047 (0.882–1.242) | 0.601 | 0.831 (0.737–0.935) | 0.002 |
| eGFR | Reference | 1.143 (0.957–1.365) | 0.139 | 0.919 (0.785–1.074) | 0.288 |
| TC | Reference | 0.959 (0.825–1.116) | 0.590 | 0.955 (0.830–1.100) | 0.525 |
| TG | Reference | 0.923 (0.771–1.103) | 0.378 | 0.897 (0.744–1.081) | 0.253 |
| Sarcopenia | Reference | 2.448 (1.208–4.960) | 0.013 | 1.945 (1.030–3.672) | 0.040 |

All continuous predictors were standardized; ORs represent the risk change per 1-SD increase.

Abbreviations: OR, odds ratios; CI, confidence interval; SD, standard deviation; PRISm, preserved ratio impaired spirometry; BMI, body mass index; Hb, hemoglobin; Alb, albumin; eGFR, estimated glomerular filtration rate; TC, total cholesterol; TG, triglycerides.
